# Publisher Correction: The impact of exercise on gene regulation in association with complex trait genetics

**DOI:** 10.1038/s41467-026-72505-6

**Published:** 2026-04-27

**Authors:** Nikolai G. Vetr, Nicole R. Gay, MoTrPAC Study Group, Nikolai G. Vetr, Nicole R. Gay, Joshua N. Adkins, Brent G. Albertson, David Amar, Mary Anne S. Amper, Jose Juan Almagro Armenteros, Euan Ashley, Julian Avila-Pacheco, Dam Bae, Ali Tugrul Balci, Marcas Bamman, Nasim Bararpour, Elisabeth R. Barton, Pierre M. Jean Beltran, Bryan C. Bergman, Daniel H. Bessesen, Sue C. Bodine, Frank W. Booth, Brian Bouverat, Thomas W. Buford, Charles F. Burant, Tiziana Caputo, Steven Carr, Toby L. Chambers, Clarisa Chavez, Maria Chikina, Roxanne Chiu, Michael Cicha, Clary B. Clish, Paul M. Coen, Dan Cooper, Elaine Cornell, Gary Cutter, Karen P. Dalton, Surendra Dasari, Courtney Dennis, Karyn Esser, Charles R. Evans, Roger Farrar, Facundo M. Fernández, Kishore Gadde, Nicole Gagne, David A. Gaul, Yongchao Ge, Robert E. Gerszten, Bret H. Goodpaster, Laurie J. Goodyear, Marina A. Gritsenko, Kristy Guevara, Fadia Haddad, Joshua R. Hansen, Melissa Harris, Trevor Hastie, Krista M. Hennig, Steven G. Hershman, Andrea Hevener, Michael F. Hirshman, Zhenxin Hou, Fang-Chi Hsu, Kim M. Huffman, Chia-Jui Hung, Chelsea Hutchinson-Bunch, Anna A. Ivanova, Bailey E. Jackson, Catherine M. Jankowski, David Jimenez-Morales, Christopher A. Jin, Neil M. Johannsen, Robert L. Newton, Maureen T. Kachman, Benjamin G. Ke, Hasmik Keshishian, Wendy M. Kohrt, Kyle S. Kramer, William E. Kraus, Ian Lanza, Christiaan Leeuwenburgh, Sarah J. Lessard, Bridget Lester, Jun Z. Li, Malene E. Lindholm, Ana K. Lira, Xueyun Liu, Ching-ju Lu, Nathan S. Makarewicz, Kristal M. Maner-Smith, D. R. Mani, Gina M. Many, Nada Marjanovic, Andrea Marshall, Shruti Marwaha, Sandy May, Edward L. Melanson, Michael E. Miller, Matthew E. Monroe, Samuel G. Moore, Ronald J. Moore, Kerrie L. Moreau, Charles C. Mundorff, Nicolas Musi, Daniel Nachun, Venugopalan D. Nair, K. Sreekumaran Nair, Michael D. Nestor, Barbara Nicklas, Pasquale Nigro, German Nudelman, Eric A. Ortlund, Marco Pahor, Cadence Pearce, Vladislav A. Petyuk, Paul D. Piehowski, Hanna Pincas, Scott Powers, David M. Presby, Wei-Jun Qian, Shlomit Radom-Aizik, Archana Natarajan Raja, Krithika Ramachandran, Megan E. Ramaker, Irene Ramos, Tuomo Rankinen, Alexander Sasha Raskind, Blake B. Rasmussen, Eric Ravussin, R. Scott Rector, W. Jack Rejeski, Collyn Z-T. Richards, Stas Rirak, Jeremy M. Robbins, Jessica L. Rooney, Aliza B. Rubenstein, Frederique Ruf-Zamojski, Scott Rushing, Tyler J. Sagendorf, Mihir Samdarshi, James A. Sanford, Evan M. Savage, Irene E. Schauer, Simon Schenk, Robert S. Schwartz, Stuart C. Sealfon, Nitish Seenarine, Kevin S. Smith, Gregory R. Smith, Michael P. Snyder, Tanu Soni, Luis Gustavo Oliveira De Sousa, Lauren M. Sparks, Alec Steep, Cynthia L. Stowe, Yifei Sun, Christopher Teng, Anna Thalacker-Mercer, John Thyfault, Rob Tibshirani, Russell Tracy, Scott Trappe, Todd A. Trappe, Karan Uppal, Sindhu Vangeti, Mital Vasoya, Elena Volpi, Alexandria Vornholt, Michael P. Walkup, Martin J. Walsh, Matthew T. Wheeler, John P. Williams, Si Wu, Ashley Xia, Zhen Yan, Xuechen Yu, Chongzhi Zang, Elena Zaslavsky, Navid Zebarjadi, Tiantian Zhang, Bingqing Zhao, Jimmy Zhen, Stephen B. Montgomery, Stephen B. Montgomery

**Affiliations:** 1 Stanford University, Stanford, CA USA; 2 Pacific Northwest National Laboratory, Richland, WA USA; 3 Joslin Diabetes Center, Boston, MA USA; 4 Icahn School of Medicine at Mount Sinai, New York City, NY USA; 5 Broad Institute, Inc, Boston, MA USA; 6 University of Iowa, Iowa City, IA USA; 7 The University of Alabama at Birmingham, Birmingham, AL USA; 8 University of Florida, Gainesville, FL USA; 9 University of Colorado Anschutz Medical Campus, Aurora, CO USA; 10 University of Missouri, Columbia, MO USA; 11 University of Michigan, Ann Arbor, MI USA; 12 Ball State University, Muncie, IN USA; 13 AdventHealth Translational Research Institute, Orlando, FL USA; 14 University of California, Irvine, CA USA; 15 University of Vermont, Burlington, VT USA; 16 The Mayo Clinic, Rochester, MN USA; 17 Georgia Institute of Technology, Atlanta, GA USA; 18 Pennington Biomedical Research Center, Baton Rouge, LA USA; 19 University of California, Los Angeles, CA USA; 20 Emory University, Atlanta, GA USA; 21 Wake Forest University, Winston-Salem, NC USA; 22 Duke University, Durham, NC USA; 23 University of Virginia School of Medicine, Charlottesville, VA USA; 24 University of Texas Health Science Center, San Antonio, TX USA; 25 University of Texas Medical Branch, Galveston, TX USA; 26 University of Kansas Medical Center, Kansas City, KS USA; 27 National Institutes of Health, Bethesda, MD USA

**Keywords:** Data integration, Gene expression, Genome-wide association studies, Transcriptomics

Correction to: *Nature Communications* 10.1038/s41467-024-45966-w, published online 01 May 2024

In this article the MoTrPAC Study Group member Facundo M. Fernández was incorrectly written as Facundo M. Fernádez. The original article has been updated.

