# Supplementary material for: The impact of exercise on gene regulation in association with complex trait genetics
Source: Nat Commun. 2024 May 1;15:3346. doi: 10.1038/s41467-024-45966-w (PMC11063075; doi:10.1038/s41467-024-45966-w)
Supplement: Supplementary file 2 — Reporting Summary [file 41467_2024_45966_MOESM2_ESM.pdf]

Corresponding author(s): Stephen Montgomery, Nikolai Vetr

Last updated by author(s): Nov 29, 2023

Reporting Summary

Nature Portfolio wishes to improve the reproducibility of the work that we publish. This form provides structure for consistency and transparency in reporting. For further information on Nature Portfolio policies, see our Editorial Policies and the Editorial Policy Checklist.

Statistics

For all statistical analyses, confirm that the following items are present in the figure legend, table legend, main text, or Methods section.

n/a	Confirmed	
☐	☒	The exact sample size (*n*) for each experimental group/condition, given as a discrete number and unit of measurement
☐	☒	A statement on whether measurements were taken from distinct samples or whether the same sample was measured repeatedly
☐	☒	The statistical test(s) used AND whether they are one- or two-sided<br>*Only common tests should be described solely by name; describe more complex techniques in the Methods section.*
☐	☒	A description of all covariates tested
☐	☒	A description of any assumptions or corrections, such as tests of normality and adjustment for multiple comparisons
☐	☒	A full description of the statistical parameters including central tendency (e.g. means) or other basic estimates (e.g. regression coefficient) AND variation (e.g. standard deviation) or associated estimates of uncertainty (e.g. confidence intervals)
☐	☒	For null hypothesis testing, the test statistic (e.g. *F*, *t*, *r*) with confidence intervals, effect sizes, degrees of freedom and *P* value noted<br>*Give P values as exact values whenever suitable.*
☐	☒	For Bayesian analysis, information on the choice of priors and Markov chain Monte Carlo settings
☐	☒	For hierarchical and complex designs, identification of the appropriate level for tests and full reporting of outcomes
☐	☒	Estimates of effect sizes (e.g. Cohen's *d*, Pearson's *r*), indicating how they were calculated

Our web collection on statistics for biologists contains articles on many of the points above.

Software and code

Policy information about availability of computer code

Data collection	No novel data was collected in this work (all data comes from landscape paper or previously published analyses)
Data analysis	R (4.0.4) was used for most data analysis, with packages:<br>fgsea: 1.16.0<br>Cairo: 1.5.15<br>DESeq2: 1.30.1<br>EnsDb.Hsapiens.v79: 2.99.0<br>EnsDb.Hsapiens.v86: 2.99.0<br>MASS: 7.3.57<br>MotrpacRatTraining6mo: 1.6.4<br>MotrpacRatTraining6moData: 1.9.1<br>arrow: 8.0.0<br>biomaRt: 2.46.3<br>caret: 6.0.92<br>circlize: 0.4.15<br>clusterProfiler: 3.18.1<br>cmdstanr: 0.3.0.9000<br>data.table: 1.14.8<br>doParallel: 1.0.17<br>dplyr: 1.1.2<br>edgeR: 3.32.1

ensembldb: 2.14.1
fitdistrplus: 1.1.8
foreach: 1.5.2
ggplot2: 3.4.2
invgamma: 1.1
jpeg: 0.1.9
jsonlite: 1.8.4
ks: 1.13.5
limma: 3.46.0
org.Hs.eg.db: 3.12.0
org.Rn.eg.db: 3.12.0
parallel: 4.0.4
plotrix: 3.8.2
posterior: 1.2.2
pracma: 2.3.8
sparklyr: 1.7.7
sparklyr.nested: 0.0.3
testit: 0.13
xlsx: 0.6.5
External software and command-line tools
plink2 (2.00a3)
GCTA (1.93.2)
MESC
LDSC (1.0.1)
Stan (2.21.0)
revTWMR
TWMR
GTEx Pipeline
Fusion
MetaXcan
GenArchDB

For manuscripts utilizing custom algorithms or software that are central to the research but not yet described in published literature, software must be made available to editors and reviewers. We strongly encourage code deposition in a community repository (e.g. GitHub). See the Nature Portfolio guidelines for submitting code & software for further information.

Data

Policy information about availability of data

All manuscripts must include a data availability statement. This statement should provide the following information, where applicable:

- Accession codes, unique identifiers, or web links for publicly available datasets
- A description of any restrictions on data availability
- For clinical datasets or third party data, please ensure that the statement adheres to our policy

This study did not generate novel data, relying instead on previously or concurrently published data. MoTrPAC PASS1B data (\url{https://doi.org/10.1101/2022.09.21.508770}) used here have been deposited at \url{https://motrpac-data.org/data-access}. Inquiries regarding access to these data should be sent to motrpac-helpdesk{at}lists.stanford.edu. Further resources are available at motrpac.org and motrpac-data.org. Where it would be difficult to re-host large datasets from GTEx \autocite{lonsdale_genotype-tissue_2013}, Open Targets \autocite{ochoa_open_2021}, and PrediXcan \autocite{barbeira_exploiting_2021}, we provide download links in the documentation of the associated code repository. Source data to generate all figures seen here are provided with this paper in the form of *.RData objects. These contain all necessary processed data to fully and quickly reproduce all paper figures using the scripts contained in \url{https://github.com/NikVetr/MoTrPAC_Complex_Traits/tree/main/scripts/figures}.

Research involving human participants, their data, or biological material

Policy information about studies with human participants or human data. See also policy information about sex, gender (identity/presentation), and sexual orientation and race, ethnicity and racism.

Reporting on sex and gender	Our analyses focused on sex-independent effects, but we otherwise referred to sex in reference to rats and as coded by GTEx.
Reporting on race, ethnicity, or other socially relevant groupings	We do not consider racial, ethnic, or other socially relevant variation in this manuscript.
Population characteristics	We use ancestry PCs from GTEx during one component of one analysis.
Recruitment	N/A
Ethics oversight	N/A

Note that full information on the approval of the study protocol must also be provided in the manuscript.

Field-specific reporting

Please select the one below that is the best fit for your research. If you are not sure, read the appropriate sections before making your selection.

☒ Life sciences ☐ Behavioural & social sciences ☐ Ecological, evolutionary & environmental sciences

For a reference copy of the document with all sections, see nature.com/documents/nr-reporting-summary-flat.pdf

Life sciences study design

All studies must disclose on these points even when the disclosure is negative.

Sample size	Sample sizes were not a researcher degree of freedom in this manuscript, as they were determined by other or previously published studies.
Data exclusions	Data were not excluded in this manuscript. Data from other work had independent exclusion criteria (eg several rat tissue samples were excluded do to contamination)
Replication	Code is provided that should allow readers to replicate all analyses performed and figures generated from publicly available data releases.
Randomization	No experimental randomization was performed here.
Blinding	No blinding was performed here. However, statistical analyses did not explicitly involve data labels, as these were converted to numeric indices. These labels were not revealed until data visualization.

Reporting for specific materials, systems and methods

We require information from authors about some types of materials, experimental systems and methods used in many studies. Here, indicate whether each material, system or method listed is relevant to your study. If you are not sure if a list item applies to your research, read the appropriate section before selecting a response.

Materials & experimental systems

n/a	Involved in the study	
☒	☐	Antibodies
☒	☐	Eukaryotic cell lines
☒	☐	Palaeontology and archaeology
☒	☐	Animals and other organisms
☒	☐	Clinical data
☒	☐	Dual use research of concern
☒	☐	Plants

Methods

n/a	Involved in the study	
☒	☐	ChIP-seq
☒	☐	Flow cytometry
☒	☐	MRI-based neuroimaging

Plants

Seed stocks	N/A
Novel plant genotypes	N/A
Authentication	N/A
