# Supplementary material for: The impact of exercise on gene regulation in association with complex trait genetics
Source: Nat Commun. 2024 May 1;15:3346. doi: 10.1038/s41467-024-45966-w (PMC11063075; doi:10.1038/s41467-024-45966-w)
Supplement: Supplementary file 3 — Supplementary Information [file 41467_2024_45966_MOESM3_ESM.pdf]

The impact of exercise on gene regulation in association with complex trait genetics

Nikolai G. Vetr[1,*], Nicole R. Gay[1], MoTrPAC Study Group, and Stephen B. Montgomery[1,†]

[1]*Stanford University, Stanford, CA, USA;*
[*]*E-mail: nikgvetr@stanford.edu*
[†]*E-mail: smontgom@stanford.edu*

SUPPLEMENTARY FIGURES

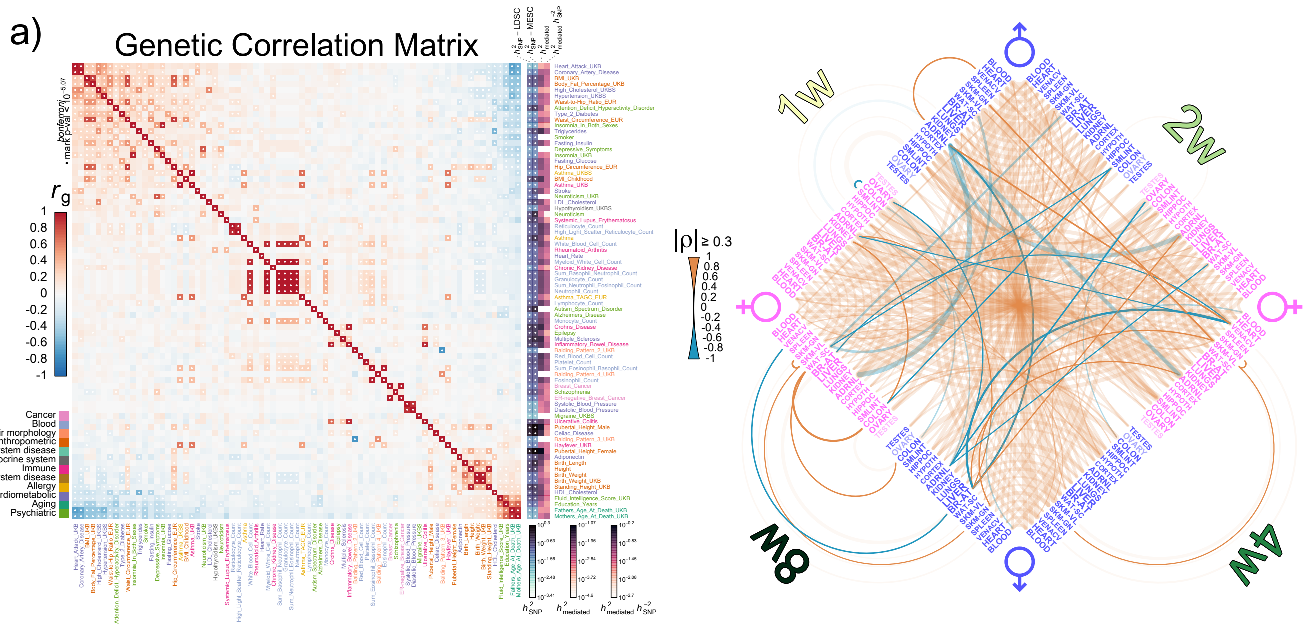

Supplementary Figure 1: Gross tissue-specific expression and genetical variation in trait expression show intermediate levels of interdependence. A high-level overview of the both the phenotypes and tissues under consideration here. In a), we visualize the additive genetic correlation matrix of the 77 traits that with SNP-heritability estimated by LDSC at $+7\sigma$ above 0. Red-blue heatmap colors correspond to estimated genetic correlations, and dots represent significant correlations that remain after Bonferroni correction targeting a FWER at $\alpha = 0.05$ (two-sided). In the right margin, we include four additional columns corresponding to each row-wise trait. From left-to-right, these represent SNP-heritability (h^2_{SNP}) estimated by LDSC, h^2_{SNP} estimated by MESC, h^2_{SNP}, expression-mediated h^2_{SNP} estimated by MESC, and the ratio of expression-mediated to total h^2_{SNP}. In b), we modify the hive plot concept to depict a Spearman correlation network of adaptive transcriptional response to long-term exercise across timepoints, sexes, and tissues. Arcs vary in thickness in proportion to the square of the magnitude of their correlation, and arcs between the same tissues have reduced opacity to better visualize relationships between different tissues. For clarity of viewing, only ρs of magnitude 0.3 or greater are plotted. Source data for this figure are provided as a Source Data file.

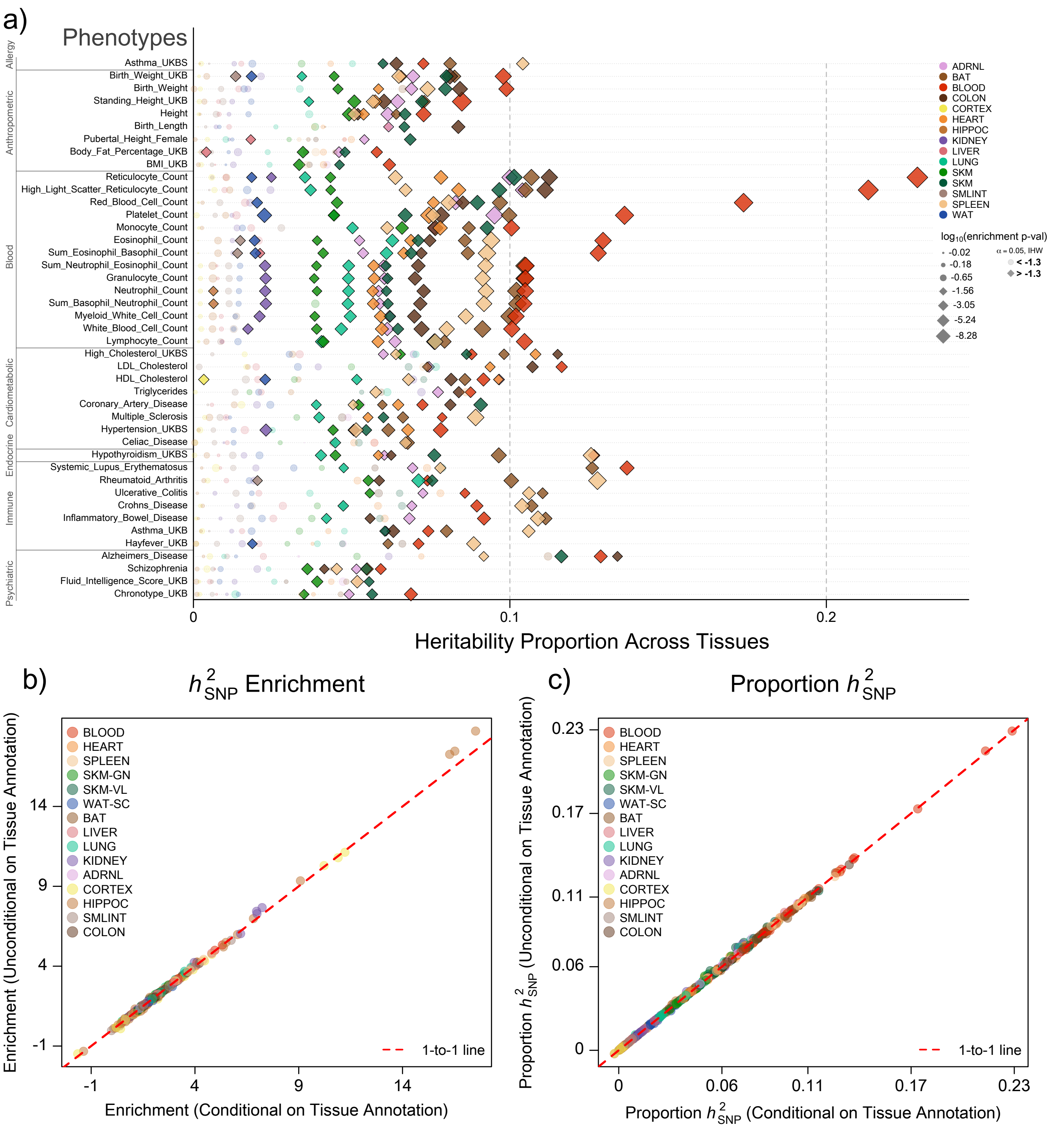

Supplementary Figure 2: Genetic variation near exercise-responsive genes captures a substantial fraction of trait heritability and is independent across tissues. Here, we visualize additional output from LDSC to accompany Fig. 3. In a), we show the proportion of total h^2_{SNP} that enrichments in Fig. 3 correspond to, marking tissues in which that total proportion was distinguishable from 0 at IHW $\alpha < 0.05$ (one-sided). In b-c), we show a scatterplot of estimated enrichments and heritability proportions (unfiltered by significance) from two parallel analyses. The horizontal axis represents estimates that are conditional on an annotation that includes all other tissue-specific annotations and a baseline annotation of 53 functional categories. The vertical axis represents estimates conditional only on the baseline annotation. Source data for this figure are provided as a Source Data file.

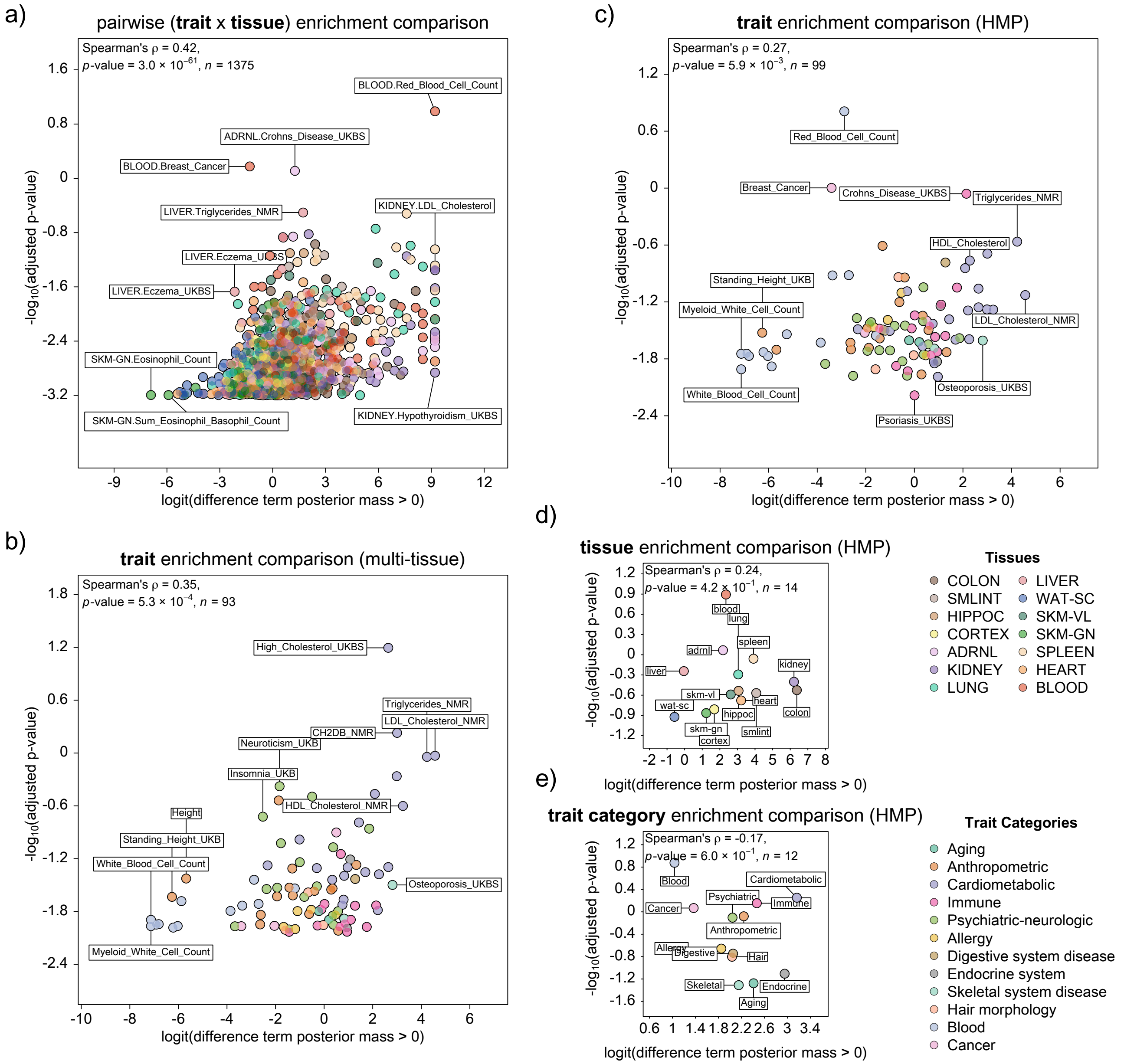

Supplementary Figure 3: Bayesian and frequentist methods broadly agree in evaluating DEG enrichment across PrediXcan output. Here, we compare enrichment results between posterior summaries from our multilevel enrichment model (horizontal axis, Fig. 5) and frequentist Gene Set Enrichment Analysis (GSEA). In a), we plot -$\log_{10}$(Bonferonni-adjusted p-values) from GSEA of Differentially Expressed Genes (DEGs) in the ranked list of unadjusted -$\log_{10}$(p-values) from PrediXcan for each trait $\times$ tissue pair. In b), we aggregate across tissues by taking only genes that are differentially expressed in three or more tissues and performing GSEA across harmonic mean p-values across the 15 matched tissues from the PrediXcan output. In c-d), we aggregate output from a) by taking harmonic mean p-values (HMP) across traits and tissues, respectively, and computing -$\log_{10}$(FWER-adjusted p-values) for plotting. In e), we take the harmonic mean of unadjusted p-values from c) across trait categories and apply the same transformation as before. Spearman's ρs are given with nominal p-values (two-sided) in the upper left corners of each plot, with sample sizes of 1,375, 93, 99, 14, and 12 for a-e, respectively. Source data for this figure are provided as a Source Data file.

Supplementary Tables

Supplementary Table 1: General Notation for Acronyms and Abbreviations: A table compiling all acronyms and abbreviations used in this manuscript.

Symbol	Interpretation
MoTrPAC	Molecular Transducers of Physical Activity Consortium
EET	Endurance Exercise Training
DE	Differential Expression
DEG	Differentially Expressed Gene
F344	Fischer 344 Inbred Rats
GTEx	Genotype-Tissue Expression project
GWAS	Genome-Wide Association Study
GCTA	Genome-wide Complex Trait Analysis
TWAS	Transcriptome-Wide Association Study
LDSC	Linkage Disequilibrium Score Regression
SNP	Single Nucleotide Polymorphism
MESC	Mediated Expression Score Regression
eQTL	Expression Quantitative Trait Loci
h^2_{SNP}	narrow-sense heritability captured by variation at SNPs
8w_F1_M1	upregulated DEGs in both males and females after 8 weeks of training
8w_F-1_M-1	downregulated DEGs in both males and females after 8 weeks of training
IHW	Independent Hypothesis Weighting
BF%	Body Fat Percentage
ADRNL	Adrenals
BAT	Brown Adipose
COLON	Colon
CORTEX	Cortex
SKM-GN	Gastrocnemius
HEART	Heart
HIPPOC	Hippocampus
HYPOTH	Hypothalmus
KIDNEY	Kidney
LIVER	Liver
LUNG	Lung
BLOOD	Blood RNA
SMLINT	Small Intestine
SPLEEN	Spleen
SKM-VL	Vastus Lateralis
WAT-SC	White Adipose

Supplementary Table 2: Intersect Enrichment Model Key: A table compiling notation used in our Intersect Enrichment model.

Symbol	Support	Dimension	Interpretation
i	$\{1, 2, ..., 15\}$	1485	tissue index
j	$\{1, 2, ..., 99\}$	1485	trait index
k	$\{1, 2, ..., 12\}$	1485	trait category index
$n_{i,j}^{DEG}$	$\mathbb{N}^0$	1485	observed # of Differentially Expressed Genes (DEG) for tissue i and trait j
$n_{i,j}^{\neg DEG}$	$\mathbb{N}^0$	1485	observed # of non-DEGs for tissue i and trait j
$y_{i,j}$	$\{1, 2, ..., n_{i,j}\}$	1485	observed # of PrediXcan hits for tissue i and trait j
f			$f : \mathbb{R} \rightarrow (0, 1)$, logit function (maps log-odds to probabilities)
$\pi_{i,j}$	$\mathbb{R}$	1485	mean log-odds of observing a PrediXcan hit for tissue i and trait j
α	$\mathbb{R}$	1	average difference in log-odds between DEGs and non-DEGs
β_i	$\mathbb{R}$	15	relative deviation to difference in log-odds between DEGs and non-DEGs for tissue i
γ_j	$\mathbb{R}$	99	relative deviation to difference in log-odds between DEGs and non-DEGs for trait j
μ_k	$\mathbb{R}$	12	relative deviation to difference in log-odds between DEGs and non-DEGs for trait category k
$\epsilon_{i,j}$	$\mathbb{R}$	1485	relative deviation to difference in log-odds between DEGs and non-DEGs for tissue i $\times$ trait j
σ^*	$\mathbb{R}_{>0}$	1	various scale hyperparameters (for normal priors)
Σ_*		15×15<br>99×99<br>1485×1485	maximum-likelihood estimated correlation matrix (bivariate probit)
λ_k	$\mathbb{R}$	12	mean log-odds of observing a PrediXcan hit for trait in category k
η_j	$\mathbb{R}$	99	mean log-odds of observing a PrediXcan hit for trait j

Supplementary Table 3: Directionality Enrichment Model Key: A table compiling notation used in our Directionality Enrichment model.

Symbol	Support	Dimension	Interpretation
i	$\{1,2,...,15\}$	1485	tissue index
j	$\{1,2,...,99\}$	1485	trait index
k	$\{1,2,...,12\}$	1485	trait category index
$n_{\text{i,j}}$	$\mathbb{N}^0$	1485	observed # of Differentially Expressed Genes (DEG) $\cap$ PrediXcan hits for tissue i and trait j
$y_{\text{i,j}}$	$\{1,2,...,n_{\text{i,j}}\}$	1485	observed # of positive associations in the set of DEGs $\cap$ PrediXcan hits for tissue i and trait j
f			$f : \mathbb{R} \to (0,1)$, logit function (maps log-odds to probabilities)
$\pi_{\text{i,j}}$	$\mathbb{R}$	1485	mean log-odds of observing a positive association for tissue i and trait j
$\vec{\mu}_{\text{j}}$	$\mathbb{R}$	99	mean log-odds of observing a positive association for trait j
S	$\mathbb{R}_{>0}$	99×99	diagonal matrix of standard deviations of trait-wise log-odds enrichments in positive effects
R	$C(\theta, G, I)\|\theta \in [0,1]$	99×99	correlation matrix of mean positive association log-odds across traits, where C is the weighted average operation
G		99×99	externally estimated SNP correlation matrix across traits
I		99×99	$j \times j$ identity matrix
θ	$\in$ [0,1]	1	weight proportion between G and I
δ	$\mathbb{R}_{>0}$	1	geometric average standard deviation of trait-wise enrichment in positive effects
γ_{k}	$\mathbb{R}$	12	multiplicative category deviation to δ for trait category k
ρ	$\mathbb{R}_{>0}$	1	geometric average standard deviation of tissue-wise enrichment in positive effects for a given trait
λ_{j}	$\mathbb{R}_{>0}$	99	multiplicative category deviation to ρ for trait j

SUPPLEMENTARY METHODS

Empirical Bayesian Estimation of Expression Variance

Full Bayesian analysis of GTEx expression variance was found to be computationally intractable, so we adopted an empirical Bayesian approximation instead. The specific steps of that analysis were:

1. Modify the gtex-pipeline using the GTEx v8 data release for each gene in each relevant tissue to use a $\log_2$(x+1) transformation instead of an inverse normal transformation.

2. Fit a linear model (via OLS) to these values independently for each gene and tissue using GTEx v8 covariates. "Regress" or "residualize" out the estimated effects of these covariates by subtracting from each individual the values corresponding to their covariates.

3. For each gene in each tissue, compute a sample variance (implicitly within-sex, as sex was a covariate in the above step).

4. For each tissue, fit an inverse gamma distribution across gene variances. Using these fitted values, update this "empirical" prior using each sample variance and sample size, separately for each sex. Fitting the inverse-gamma separately for each sex would have yielded fully sex-specific estimates, but we estimated pooled hyperparameters and unpooled posteriors to approximate regularization of each sex-specific variance towards a pooled mean variance.

5. Compute the expectation of these sex-, tissue-, and gene-specific posterior distributions as an estimate of total phenotypic variance stratified along the above axes.
